# Immunosuppression by viral N proteins

**DOI:** 10.18632/oncotarget.18597

**Published:** 2017-06-22

**Authors:** Jidang Chen, Hinh Ly

**Affiliations:** Department of Veterinary and Biomedical Sciences, University of Minnesota, Twin Cities, College of Veterinary Medicine, Saint Paul, MN, USA

**Keywords:** arenavirus, SARS-CoV, influenza virus, interferons, immune evasion

Arenaviruses and Coronaviruses (CoV) are enveloped RNA viruses. The nucleoproteins (N) of these viruses (termed nucleocapsid in CoV) play a key role in the formation of the viral ribonucleoprotein (RNP) complexes and in causing type I interferon (IFN) suppression. The primary function of the N proteins of CoV, such as the severe acute respiratory syndrome coronavirus (SARS-CoV), and of arenaviruses, such as Lassa virus that can cause severe and lethal hemorrhagic fever infections in humans, is to package the viral single stranded genomic RNA(s) into RNP complexes [1, 2]. Another important function of these proteins is to suppress type I interferons (IFNs) in infected cells, the molecular mechanisms of which have only recently been elucidated and will be discussed in this article.

The host innate immune system presents a significant barrier and defense against viruses. The host pattern recognition receptors (PRRs), including retinoic acid-inducible gene I (RIG-I), melanoma differentiation-associated protein 5 (MDA5) and laboratory of genetics and physiology 2 protein (LGP2), can specifically recognize virus-specific components, such as RNA, DNA or glycoproteins. Following virus recognition by the PRRs, important cellular signaling pathways are activated to lead to production of cytokines, chemokines and type I IFNs, which play a critical role in the eradication of the virus. Arenaviruses and CoV both have evolved unique mechanisms to evade recognition by PRRs, which lead to type 1 IFN inhibition.

Immune stimulatory dsRNA generated during virus replication is important for RIG-I recognition and subsequently mediating type-I IFN production. We have previously discovered that arenaviruses encode a 3'-5'exoribonuclease function in the C-terminal domain of the N protein (NP) that acts as a type I IFN-antagonist [3]. Arenavirus NP exoribonuclease, a RNase member of the DEDDh superfamily, degrades dsRNA which is the important signal for RIG-I activation. We have shown that recombinant arenaviruses carrying NP RNase-defective mutations induce strong IFN responses to inhibit virus replication early in infection *in vivo* through the activation of the RIG-I pathway [3].

Coronaviruses are the only other family of viruses known to encode the DEDDh exoribonuclease in the N-terminal domain of their non-structural protein 14 (nsp14), which has been attributed to its important proofreading role during viral RNA genome replication [4]. It has recently been shown that nsp14 exoribonuclease can also inhibit type I IFN production [5]. Recombinant viruses with mutations in one of the zinc-finger motifs of the nsp14 exoribonuclease domain of the transmissible gastroenteritis coronavirus (TGEV) were found to only mildly affect genome replication and transcription, but they appeared to result in less dsRNA accumulation in virus-infected cells. The reduction in dsRNA levels correlates with a decrease in the levels of type I IFNs and of some representative interferon-stimulated genes (ISGs). Taken together, coronaviral nsp14 exoribonuclease, similar to that of the arenaviral NP exoribonuclease, has the potential to degrade immune stimulatory dsRNA during virus replication and thereby suppress type I IFN production.

The N protein of CoV has recently been found to employ a different way to mediate suppression of type 1 IFN production. Hu et al. [6] showed that the C-terminus of the SARS-CoV N protein could bind to the SPRY domain of the cellular TRIM25 E3 ubiquitin ligase. This tripartite motif protein 25 (TRIM25) E3 ligase plays important role in post-translational modification of the N terminal caspase recruitment domains (CARDs) of RIG-I by ubiquitination [7]. The interaction between the C-terminus of N protein of SARS-CoV with the SPRY domain of the TRIM25 E3 ubiquitin ligase interferes with the association between TRIM25 and RIG-I and thereby inhibiting TRIM25-mediated RIG-I ubiquitination and activation and type 1 IFN production [6]. Similarly, Gack et al. have reported that influenza A virus (IAV) contains a novel domain in its NS1 protein that can also block TRIM25 multimerization and RIG-I CARD domain ubiquitination via the interaction between IAV NS1 protein with the coiled-coil domain of TRIM25 [7]. Taken together, the molecular mechanism of inhibition of type 1 IFN production via RIG-I's ubiquitination by direct protein-protein interaction appears to have been exploited by different viruses that include but are not necessarily limited to SARV-CoV and IAV. Therefore, an in-depth understanding of the conserved molecular mechanisms of innate immune evasion by different viruses may lead to the development of novel broad-spectrum antivirals against many medically significant human viruses.

## References

[1] Chang CK (2014). Antiviral Research.

[2] Pinschewer DD (2003). Journal of Virology.

[3] Huang Q (2015). Journal of Virology.

[4] Sevajol M (2014). Virus Research.

[5] Becares M (2016). Journal of Virology.

[6] Hu Y (2017). Journal of Virology.

[7] Gack MU (2009). Cell Host & Microbe.

